# Multi-channel GAN–based calibration-free diffusion-weighted liver imaging with simultaneous coil sensitivity estimation and reconstruction

**DOI:** 10.3389/fonc.2023.1095637

**Published:** 2023-02-08

**Authors:** Jun Lyu, Yan Li, Fuhua Yan, Weibo Chen, Chengyan Wang, Ruokun Li

**Affiliations:** ^1^ School of Computer and Control Engineering, Yantai University, Yantai, Shandong, China; ^2^ Department of Radiology, Ruijin Hospital, Shanghai Jiao Tong University School of Medicine, Shanghai, China; ^3^ College of Health Science and Technology, Shanghai Jiao Tong University School of Medicine, Shanghai, China; ^4^ Philips Healthcare (China), Shanghai, China; ^5^ Human Phenome Institute, Fudan University, Shanghai, China

**Keywords:** multi-channel generative adversarial network, calibration-free, simultaneous estimation, coil sensitivity estimation, DWI reconstruction, liver

## Abstract

**Introduction:**

Diffusion-weighted imaging (DWI) with parallel reconstruction may suffer from a mismatch between the coil calibration scan and imaging scan due to motions, especially for abdominal imaging.

**Methods:**

This study aimed to construct an iterative multichannel generative adversarial network (iMCGAN)-based framework for simultaneous sensitivity map estimation and calibration-free image reconstruction. The study included 106 healthy volunteers and 10 patients with tumors.

**Results:**

The performance of iMCGAN was evaluated in healthy participants and patients and compared with the SAKE, ALOHA-net, and DeepcomplexMRI reconstructions. The peak signal-to-noise ratio (PSNR), structural similarity index measure (SSIM), root mean squared error (RMSE), and histograms of apparent diffusion coefficient (ADC) maps were calculated for assessing image qualities. The proposed iMCGAN outperformed the other methods in terms of the PSNR (iMCGAN: 41.82 ± 2.14; SAKE: 17.38 ± 1.78; ALOHA-net: 20.43 ± 2.11 and DeepcomplexMRI: 39.78 ± 2.78) for b = 800 DWI with an acceleration factor of 4. Besides, the ghosting artifacts in the SENSE due to the mismatch between the DW image and the sensitivity maps were avoided using the iMCGAN model.

**Discussion:**

The current model iteratively refined the sensitivity maps and the reconstructed images without additional acquisitions. Thus, the quality of the reconstructed image was improved, and the aliasing artifact was alleviated when motions occurred during the imaging procedure.

## 1 Introduction

Diffusion-weighted imaging (DWI) has been widely used in the diagnosis of tumors Kim et al. (1); Zarghampour et al. (2); Tang and Zhou (3), stroke Sartoretti et al. (4); Nazari-Farsani et al. (5); Nagaraja et al. (6), and trauma Wortman et al. (7); Lindsey et al. (8); Mohammadian et al. (9). Single-shot echo-planar imaging (SS-EPI) Baliyan et al. (10) is frequently used in DWI due to its rapid acquisition speed. But the readout duration of the SS-EPI is relatively long. Therefore, to acquire an acceptable resolution, the echo train length should be increased. But it results in T2* blurring artifacts and geometric distortion Farzaneh et al. (11); Liu et al. (12), especially in abdominal imaging.

Parallel imaging (PI) can be applied to the SS-EPI to eliminate these untoward effects. In abdominal DWI, an extra coil calibration scan with the turned-off diffusion gradients (b = 0 , b0) is employed to extract the required PI calibration information. Depending on the use of calibration information, the PI can be categorized into three groups, explicit calibration, auto-calibration, and calibration-free approaches. Explicit-calibration approaches, including sensitivity-encoding (SENSE) Pruessmann et al. (13), rely on coil sensitivities that are estimated using a separate calibration scan before or after the accelerated imaging, which prolongs the total scan time. Furthermore, a mismatch between calibration and imaging due to the interscan motion causes significant artifacts in reconstructed images Blaimer et al. (14), which is a critical problem in abdominal imaging. The auto-calibration either obtains the sensitivity information by interpolating kernels gained from the calibration areas or by using its spatial reconstruction to overcome challenges associated with explicit calibration. Algorithms included generalized auto-calibrating partially parallel acquisitions GRAPPA Griswold et al. (15), SPIRiT Lustig and Pauly (16), L1-SPIRiT Murphy et al. (17), and ESPIRiT Uecker et al. (18). Since these methods failed when the acceleration factors were high, several other algorithms, such as JSENSE Ying and Sheng (19), IRGN-TV Uecker et al. (20), self-feeding sparse SENSE Huang et al. (21), and Sparse BLIP She et al. (22) were used to simultaneously estimate the image and the sensitivity. Nevertheless, these joint estimating methods are sensitive to initialization. Additionally, these auto-calibration methods cannot be used for DWI because PI requires uniformly sampled *k*-space for SS-EPI. If used, it results in insufficient autocalibration signals (ACSs). Therefore, an additional coil calibration scan and acquisition are required, which bring the same effect as explicit calibration Yi et al. (23). To overcome these drawbacks, several calibration-free strategies were developed recently. The SAKEShin et al. (24) (Simultaneous Auto-calibration and k-space Estimation), P-LORAKS Haldar and Zhuo (25) (Parallel-imaging LOw-RAnking matrix modeling of local *k*-space neighborhoods), and ALOHA Jin and Ye (26) (Annihilating filter-based LOw-rank HAnkel matrix) use structured low-rank matrix completion to recover a full *k*-space from incomplete data. Besides, CLEAR Trzasko and Manduca (27) (Calibration-free Locally low-rank EncourAging Reconstruction) exhibits the property of low-rankness in the image domain. These methods are capable of producing state-of-the-art reconstruction performance. However, the algorithms have three main shortcomings. First, they are limited by their computational complexity which requires a long time for iterative calculations; Second, the CS algorithms require access to the *k*-space data, so quality improvement in the image domain using post-processing techniques is not possible; Third, they cannot rectify the artifacts due to uniformly under-sampling.

Recently, several deep learning-based calibrations-free algorithms Kwon et al. (28); Zhang et al. (29); Han et al. (30); McRobbie et al. (31); Arvinte et al. (32); Hu et al. (33) were successfully applied to the PI to shorten the reconstruction time and prevent the separate coil-calibration procedure. Kwon et al. Kwon et al. (28) adopted a learning-based design using the multilayer perceptron (MLP) training algorithm to reconstruct the images from subsampled data. Line-by-line processing of the learned MLP architecture reduced the aliasing artifacts. Zhang et al. Zhang et al. (29) developed a multi-channel generative adversarial network for MRI reconstruction without using sensitivity maps. The images were generated from under-sampled multi-channel raw data directly by estimating missing data with the trained network. The deep complex MRI Wang et al. (34) was used to develop end-to-end learning and it directly reconstructed the multi-channel images without explicitly using coil sensitivity maps. Deep J-Sense Arvinte et al. (32) unrolls an alternating optimization to jointly solve for the image and sensitivity map kernels directly in k-space for parallel MRI reconstruction. RUN-UP Hu et al. (33) utilize an unrolled network with U-Nets alternating in k-space and image space as deep priors to achieve fast and high-quality multi-shot DWI reconstruction. Furthermore, Han et al. Han et al. (30) proposed ALOHA-net, a fully data-driven deep learning algorithm for k-space interpolation, connecting the ALOHA and deep learning. Nevertheless, most of the aforementioned studies were based on variable density sampling for image reconstruction. None of the elucidations considered reconstructing DWIs acquired with uniform under-sampling. Also, the uniform under-sampling in Cartesian trajectory resulted in wrap-around artifacts (also called aliasing artifacts) in MR images McRobbie et al. (31). Further, the obtained sensitivity maps also contained aliasing artifacts with a low signal-to-noise ratio.

In this study, an iterative multi-channel generative adversarial network (iMCGAN)-based framework was constructed for joint sensitivity map and calibration-free image reconstruction in multi-channel EPI-based abdominal DWI with uniform subsampling. First, the iMCGAN reconstructed the multi-channel uniform under-sampling abdominal DW data and coil sensitivity maps. Second, it effectively eliminated the mismatch between the coil calibration scan and the main scan due to the inter-scan motion.

## 2 Theory

### 2.1 General image reconstruction

The forward measurement model is formulated as follows for multi-coil acquisition:


(1) $$y_{u}=\text{MSFx,}$$

where *M* denotes an under-sampling mask, *F* indicates the Fourier Transform operator, *S* denotes the coil sensitivity map, is the acquired under-sampled *k*-space data, and *x* denotes the images that are reconstructed from fully sampled *k*-space. In calibration-free image reconstruction, the coil sensitivity maps are usually estimated using the following equation:


(2) $$S^{i}=\frac{x^{i}}{\sqrt{\sum_{i}{\left|x^{i}\right|}^{2}}}$$

where the x^*i*^ represents the image that is acquired from the *i* ^th^ coil while S^*i*^ represents the *i* ^th^ coil sensitivity map. The optimization problem is expressed as minimized reconstruction error to solve the ill-posed inverse problem:


(3) $$\begin{array}{cc} \hat{x}=\arg\underset{\hat{x}}{\min} & {\left\|\hat{x}-R_{x}\left(x_{u};\theta_{x}\right)\right\|}_{2}^{2} \end{array}+\lambda {\left\|y_{u}-\text{MFS }\hat{x}\right\|}_{2}^{2},$$

where the $\hat{x}$ is the alias-free image to be reconstructed, *R_x_* is the network prior with parameters *θ*_*x*_ and input of the zero-filled (ZF) image xu=F^-1^(y_u_). The second term in Eq. 3 is the data consistency term, which is implemented in the generator of iMCGAN as a DC block. The *k*-space data in M is replaced by the corresponding data from during each iteration. The conditional GANs are also included in MRI reconstruction. The GAN is composed of a generator network *G* and a discriminator network *D*. The generator *G* aims to generate data that could fool the discriminator *D*. The discriminator *D* distinguishes the true data from the output of the generator. Therefore, the conditional GAN loss is incorporated into MRI reconstruction instead of using a CNN.


(4) $$\underset{G}{\min}\underset{D}{\max}L_{adv}(G,D)=E_{x∼P_{train}(x)}\left[\text{log }D(\text{x})\right]+E_{x_{u}∼P_{G}(x_{u})}\left[-\log D(G({\text{x}}_{u}))\right].$$

Upon learning, the generator yields the corresponding de-aliasing reconstruction $\hat{x}$ , which is fed to the discriminator. The objective is to keep the training until the discriminator distinguishes the de-aliasing reconstruction $\hat{x}$ from the fully sampled ground truth x. Any inaccuracy in sensitivity map estimation propagates to the image reconstruction process during the network training.

### 2.2 Proposed iMCGAN

**Figure 1:**
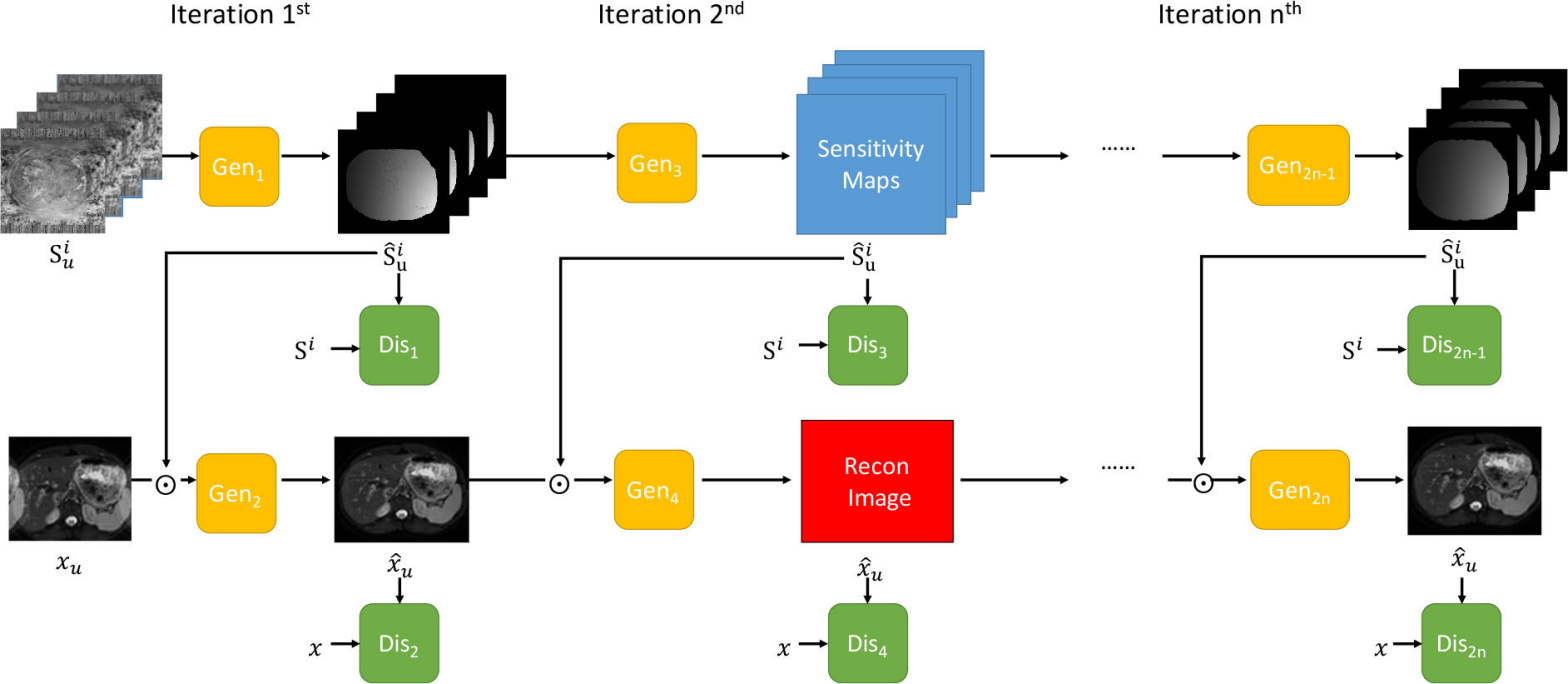
The overall framework of the proposed iterative multi-channel GAN. Gen represents the generator and Dis indicates the discriminator. Three iterations are included.


A novel iterative multi-channel GAN (iMCGAN)-based framework is constructed for joint sensitivity map estimation and calibration-free DWI reconstruction to improve the accuracy. Starting with an initial sensitivity map estimation using Eq. 2, updating the image and the coil sensitivity maps is alternated using two GANs. First, the image is fixed to update the sensitivity map estimation. One generator (GAN_2*n*-1_ in **Figure 1**) is trained by minimizing the following equation:

(5) $$L_{csm}=L_{GEN}(S_{u})+\alpha L_{\text{iMAE}}(S_{u})+\beta L_{\text{fMAE}}(S_{u}).$$

where, α and β are the hyperparameters that controlled the trade-off between functions. The mean absolute error (MAE) loss is represented as


(6) $$\underset{\theta_{G}}{\min}L_{\text{iMAE}}\left(\theta_{G}\right)=\sum_{i=1}^{C}{\left\|S^{i}-{\hat{S}}_{u}^{i}\right\|}_{1},$$

(7) $$\underset{\theta_{G}}{\min}L_{\text{fMAE}}\left(\theta_{G}\right)=\sum_{i=1}^{C}{\left\|{ℑ}^{i}-{\hat{ℑ}}_{u}^{i}\right\|}_{1},$$

where *S* ^i^ and ${\hat{S}}_{u}^{i}$ represented the full-sampled and under-sampled coil sensitivity map calculated from Eq. 2, *ℑ* ^*i*^ and ${\hat{ℑ}}_{u}^{i}$ are the Fourier transformation format of and ${\hat{S}}_{u}^{i}$ The *C* is the total number of receiver coils. The adversarial loss of the generator was as follows:


(8) $$\underset{\theta_{G}}{\min}L_{\text{GEN}}\left(\theta_{G}\right)=-\log\left(D_{\theta_{D}}\left(G_{\theta_{G}}\left(S_{u}\right)\right)\right)$$

Once the generator of GAN_2*n*-1_ was trained based on the , any new is fed to it to yield the de-aliasing sensitivity map reconstruction.

Then, the sensitivity maps for the previous iteration is fixed to update the image. The remaining reconstruction is implemented using the PIC-GAN Lv et al. (35) framework. In the PIC-GAN, the estimated coil sensitivity map ${\hat{S}}_{u}$ and the combined reconstructed image ${\hat{x}}_{u}$ are simultaneously passed to the GAN_2*n*_. This network is trained by minimizing the following equation:


(9) $$L_{\text{image}}=L_{\text{GEN}}(x_{u})+\alpha L_{\text{iMAE}}(x_{u})+\beta L_{\text{fMAE}}(x_{u})$$

where, α and β are hyperparameters that control the trade-off functions. The three loss functions were written as:


(10) $$\underset{\theta_{G}}{\min}L_{\text{iMAE}}\left(\theta_{G}\right)=\sum_{i=1}^{C}{\left\|x^{i}-{\hat{S}}_{u}^{i}{\hat{x}}_{u}\right\|}_{1},$$

(11) $$\underset{\theta_{G}}{\min}L_{\text{fMAE}}\left(\theta_{G}\right)=\sum_{i=1}^{C}{\left\|y^{i}-F{\hat{S}}_{u}^{i}{\hat{x}}_{u}\right\|}_{1},$$

(12) $$\underset{\theta_{G}}{\min}L_{\text{GEN}}\left(\theta_{G}\right)=-\log\left(D_{\theta_{D}}\left(G_{\theta_{G}}\left(x_{u}\right)\right)\right).$$

The followings are considered for the separation of image-domain and *k*-space losses. The *L*_iMAE_ (*θ_G_*) term removes the aliasing artifacts of the reconstruction and results in the image domain, which is necessary for spatial recovery of aliasing-free images. The *L*_fMAE_ (*θ_G_*) term guarantees that the reconstruction produces corresponding under-sampled image that matches the acquired under-sampled *k*-space measurements. Additionally, it minimizes the difference between the interpolated *k*-space data and the GT in our setting. The GAN models are difficult to be trained because they need to be trained alternatively Quan et al. (36) 134. Therefore, refinement learning Yang et al. (37) is incorporated to stabilize the training of the proposed model. Therefore, is used. The ${\hat{x}}_{u}=G_{\theta_{G}}(x_{u})+x_{u}$ generated information is not acquired, which significantly reduces the complexity of the model.

### 2.3 Architecture of iMCGAN

**Figure 2:**
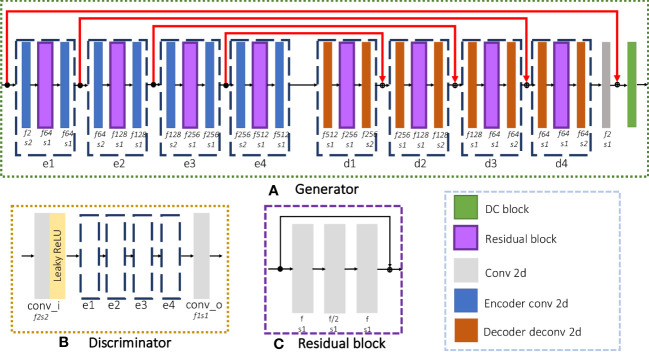
The detailed architecture of each GAN consisted of a **(A)** Generator and a **(B)** Discriminator and **(C)** the residual block used in the network.

The proposed iMCGAN is presented in this section. Three iterations are used to obtain both coil sensitivities and high-quality DW images from the under-sampled multi-channel data. In the constructed iteration architecture, each iteration includes two GANs, sharing the same architecture. As shown in **Figure 2A**, the generator is composed of an encoding path and a symmetric decoding path. The encoder modules (e1, e2, e3, and e4) are fed up with a 4D tensor input and perform the 2D convolution with a filter size of 3×3. The number of feature maps f and stride s is indicated at the bottom of each block. The decoder modules (d1, d2, d3, and d4) perform transpose convolution. As illustrated in **Figure 2C**, the residual block of the encoder and decoder consists of three convolution layers that aim to increase the depth of the generator and discriminator. The final reconstruction is obtained by inputting the ZF images and outputting aliasing free images through the generator, which is similar to many deep learning-based procedures. The architecture of the discriminator, shown in **Figure 2B**, is the same as the encoding path of the generator. In the present study, the real and imaginary components of the complex data were fed into separate channels during the network training.

## 3 Materials and methods

### 3.1 Image acquisition

This study was approved by the Institutional Review Board. The written informed consent documents were obtained from 106 healthy participants and 10 patients who had hepatic tumors. Participants underwent scans using a 3T Philips Ingenia MRI system (Philips Healthcare, Best, the Netherlands) containing an eight-channel coil. Two breath-hold DW imaging acquisitions were done for *b* values of 0 and 800 with the following parameters: single-shot spin-echo EPI; repetition time (TR)/echo time (TE) of 4000/55 ms; matrix size of 336×336; field of view (FOV) of 360×360 mm; the number of slices of 30; slice thickness of 6 mm; bandwidth of 2548 Hz/Px. The data were split into training and testing sets (**Table 1**). Additionally, a reference-free ghost correction algorithm Skare et al. (38) was implemented to remove the N/2 ghosting artifact. The acquisition time was about 12 s for each *b* value.

**Table 1:** Number of images used for training and test sets.

Examination	Participants	Number of slices
Training	85 healthy participants	2550
Test	21 healthy participants	630
	10 patients with tumors	300

### 3.2 Implementation details

The iMCGAN model was compared with three state-of-the-art methods including SAKE Shin et al. (24), ALOHA-net Han et al. (30), and DeepcomplexMRI Wang et al. (34). Three acceleration factors (AF, 2, 3, and 4) with uniform under-sampling patterns were validated. The recommended parameter settings were implemented in the corresponding studies. Besides, the sensitivity map was calculated using ESPIRiT Uecker et al. (18) with 24 ACS lines for the SENSE reconstruction. The reconstruction was performed on a workstation using specifications of Intel (R) Xeon (R) CPU E5-2698 v4 @ 2.20 GHz with 256 GB RAM and an NVIDIA GV100GL (Tesla V100 DGXS 32GB) graphics processing unit. The iMCGAN was implemented in Python with a TensorFlow backend. The maximum number of iterations 170 was set to 500 epochs during the training process. Based on previous studies Yang et al. (37); Quan et al. (36), 171 it was set at α=1 and *β*=10 with the Adam optimizer to balance the values of different loss terms into similar scales. The batch size was 16 and the initial learning rate was set at which was decreased monotonically for the training. The loss was calculated for each iteration and the parameters were independent in this study. Each sub-unit of GAN in the iMCGAN was trained separately. The training time for a sensitivity estimation of the GAN was about 2 hours and image reconstruction for the GAN was 2.5 hours, while the testing time was 0.02 ms and 0.01 ms for coil sensitivity map and image, respectively.

### 3.3 Quantitative evaluation

The reconstruction parameters were quantified using three metrics: peak signal-to-noise ratio (PSNR), structural similarity index measure (SSIM), and root mean squared error (RMSE). A paired Wilcoxon signed-rank test compared the PSNR, SSIM, and RMSE measurements between different approaches. A *P*-value of<0.05 indicated a statistically significant difference. The ADC maps were calculated from the images using the following equation:


(13) $$ADC=-\text{log}\left(I_{D},I_{0}\right)b$$

where I_*D*_ and I_0_ represented the signal intensities for b = 800 s/mm^2^ DWI and b = 0 s/mm^2^, respectively. The parameters derived from the histogram of the ADC maps included ADCmean, ADCmedian, skewness, and kurtosis.

## 4 Results

**Figure 3:**
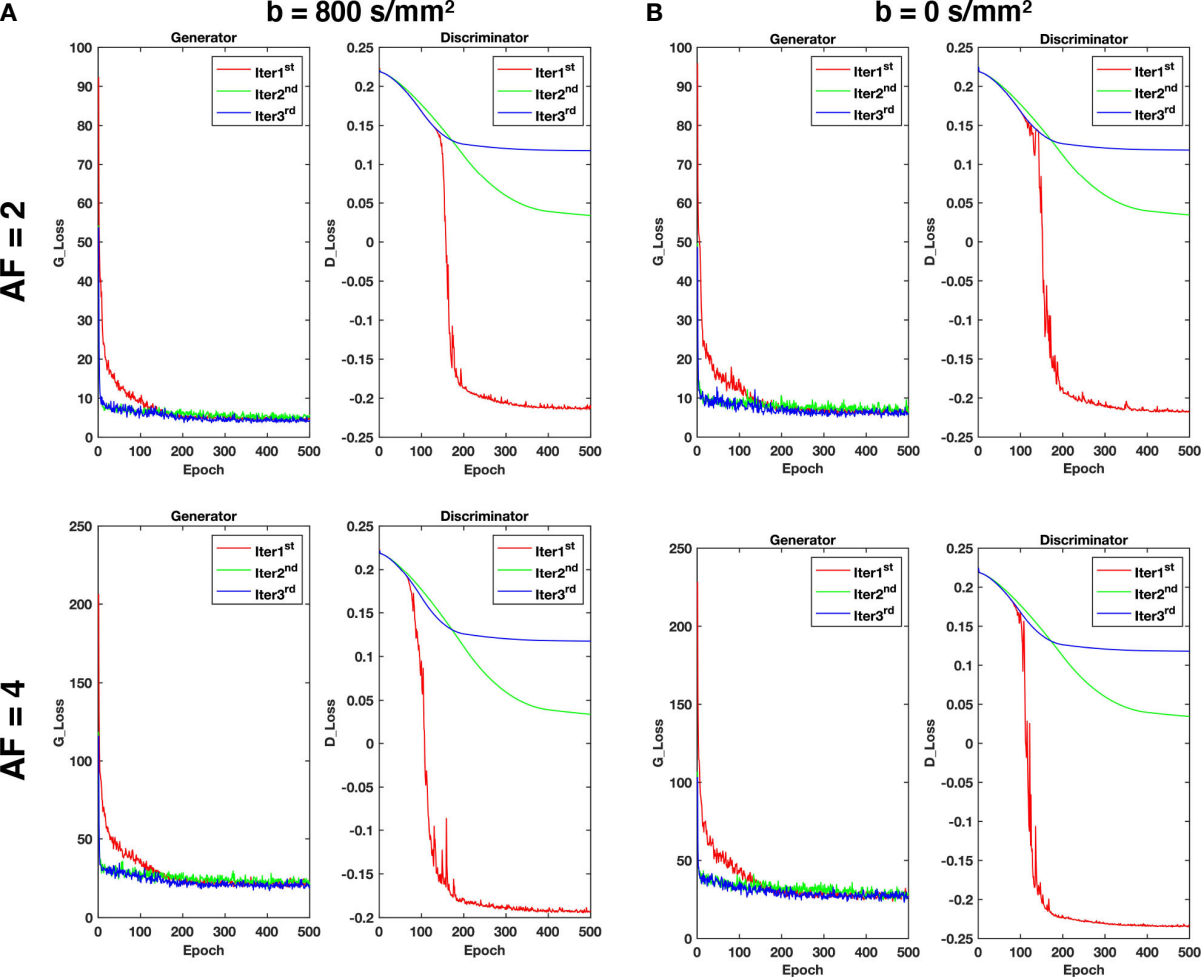
The loss curves of the generator and discriminator during training with different acceleration factors for **(A)** b = 800 s/mm^2^ and **(B)** b = 0 s/mm^2^.

As shown in **Figure 3**, the value of the loss is large and decreases very rapidly during the Iter 1^*st*^ stage, and then it is relatively stable after 200 epochs. In the Iter 2^*nd*^ and Iter 3^*rd*^ stages, the value of the loss remains basically unchanged. Therefore, it is reasonable that the model has converged in three iterations and it can achieve better reconstruction performance.

**Figure 4:**
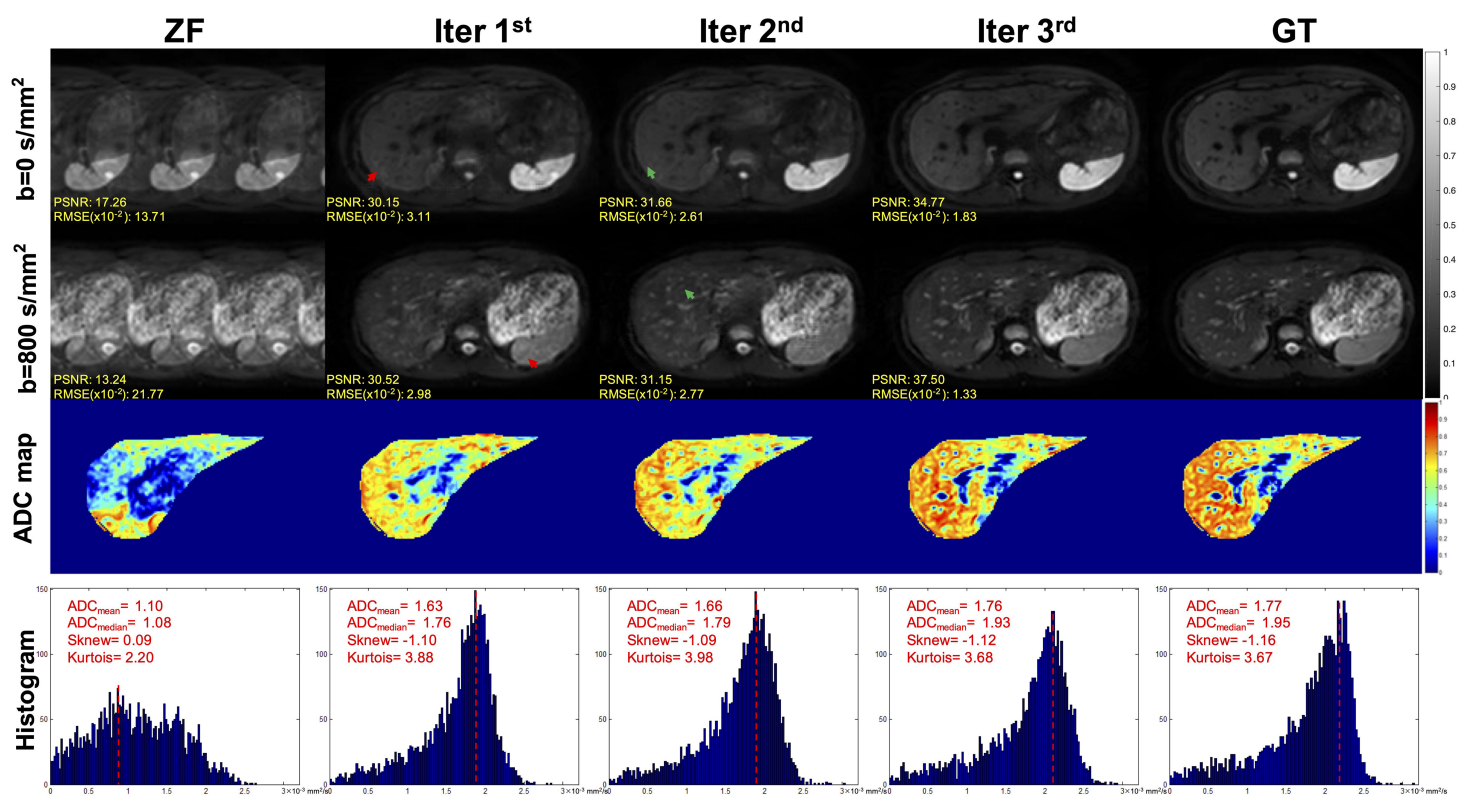
Iterative iMCGAN reconstruction at the AF of 4. The PSNR and RMSE values are shown in the corner. For a fair visual comparison, the images were normalized to [0, 1]. The selected whole-liver ADC maps and the corresponding ADC histograms are shown in the third and fourth rows. The histogram parameters are displayed in the upper left corner.

The DWI and ZF images showed prominent aliasing artifacts for b = 0 s/mm^2^ and b = 800 s/mm^2^ (**Figure 4**). Some blurring and residual artifacts still existed, as indicated by the red arrows in the first iteration (Iter 1^st^) image. The GAN considered the result of Iter 1^st^ and reconstructed a better image with very few artifacts in the second iteration (Iter 2^nd^). Nevertheless, some small vessels were missing, and the vessel edges were not clear on the image (indicated by the green arrows). The GAN took the result of Iter 2^nd^ into account and reconstructed a clear image with almost no artifacts in the third iteration (Iter 3^rd^). The measurements obtained from the liver on ADC maps were lower in the Iter 1^st^ and Iter 2^nd^ compared with the Iter 3^rd^. As the number of iterations increased, the ADC value of the Iter 3^rd^ was closer to that of the GT. The histogram of ZF was different compared with the GT, which was due to the under-sampling in the *k*-space and thus the aliasing artifacts in the spatial domain. The histogram parameters of the Iter 3^rd^ (ADCmean = 1.76; ADCmedian = 1.93; Sknew = -1.12; kurtosis = 3.68) were consistent with those of the GT (ADCmean = 1.77; ADCmedian = 1.95; Sknew= -1.16, kurtosis = 3.67).

**Figure 5:**
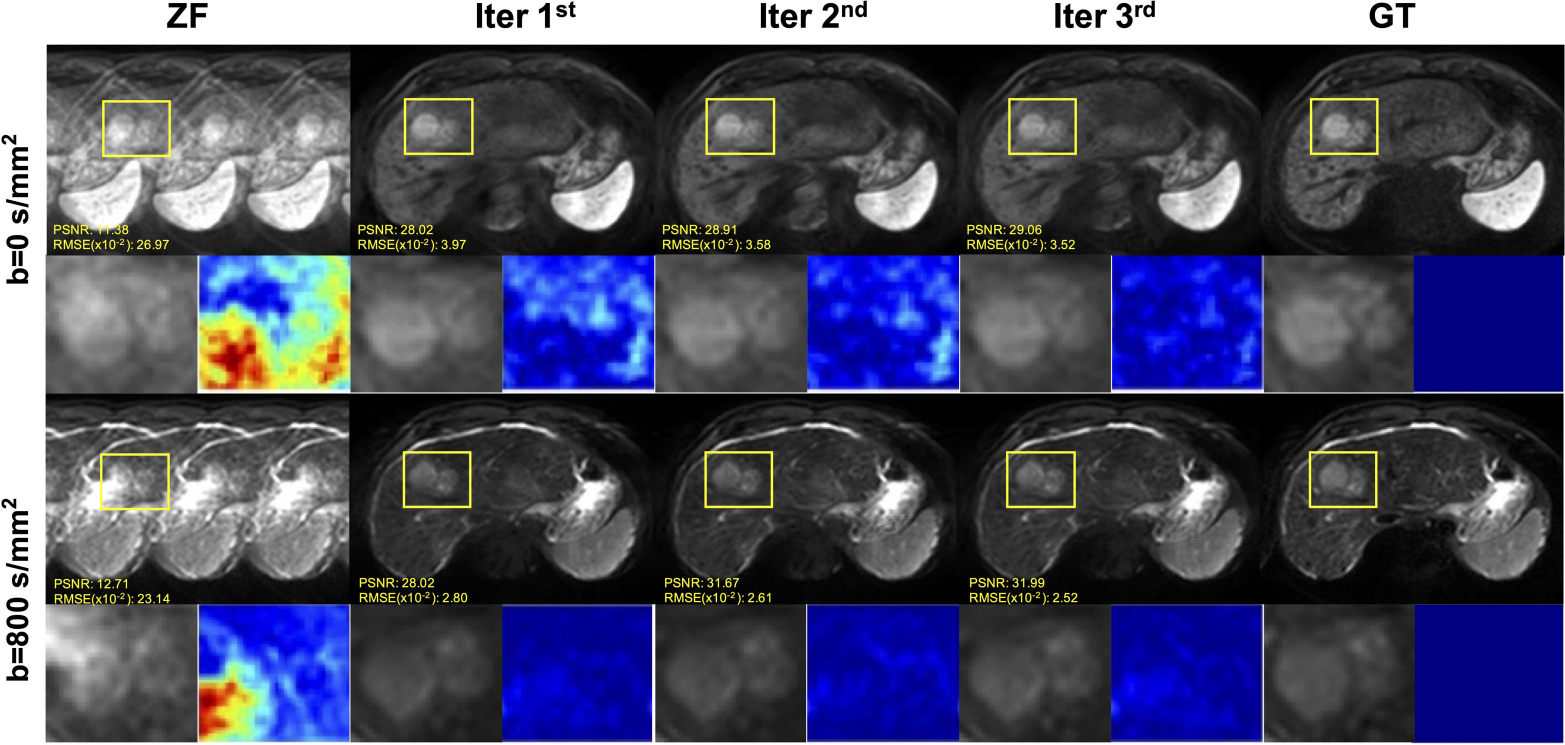
The iterative iMCGAN reconstruction with the tumor at the AF of 4. The PSNR and RMSE values are shown in the corner. For a fair visual comparison, the images were normalized to [0, 1]. The enlarged and different images with the tumor are illustrated with the reconstruction.

The iterative iMCGAN reconstruction resulted in the tumor at AF of 4 (**Figure 5**). Although all the iMCGAN reconstruction provided good results the tumor edges and textures were very accurate in the Iter 3rd image as shown in the enlarged and other images. Also, based on the PSNR and RMSE (×10) values, the Iter 3rd produced the best quantitative values (PSNR = 29.06, RMSE = 3.52 for b = 0, and PSNR = 31.99, RMSE = 2.52 for b = 800 s/mm^2^).

**Figure 6:**
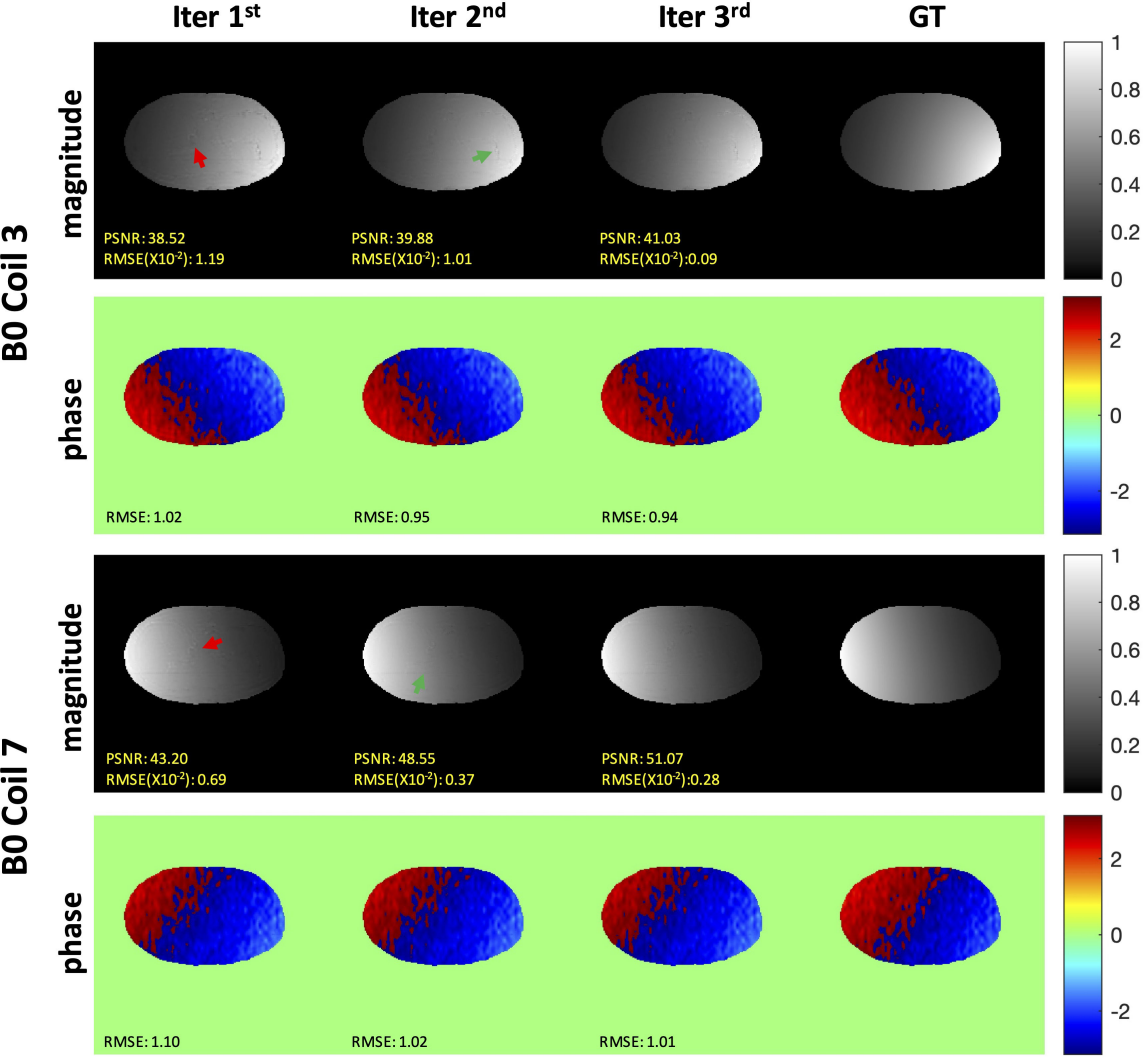
The representative iterative sensitivity map estimation of the two coil units. The 1st and 3rd rows show the magnitude images with the sensitivity profiles. The 2nd and 4th rows indicate phase images with sensitivity profiles.

The representative iterative reconstruction estimated the sensitivity maps of coils 3 and 7 at the AF of 2 (**Figure 6**). Iter 1 (a) showed a spatial artifact that was obvious at the liver edge and on the vessel area (red arrows). Iter 2 (b) showed fewer artifacts (green arrows). The map estimated by the Iter 3 (c) visually agreed with the smooth GT (d) with no artifacts. The quality metric values of both magnitude and phase indicated that the iMCGAN improved the sensitivity maps through iterations.

**Figure 7:**
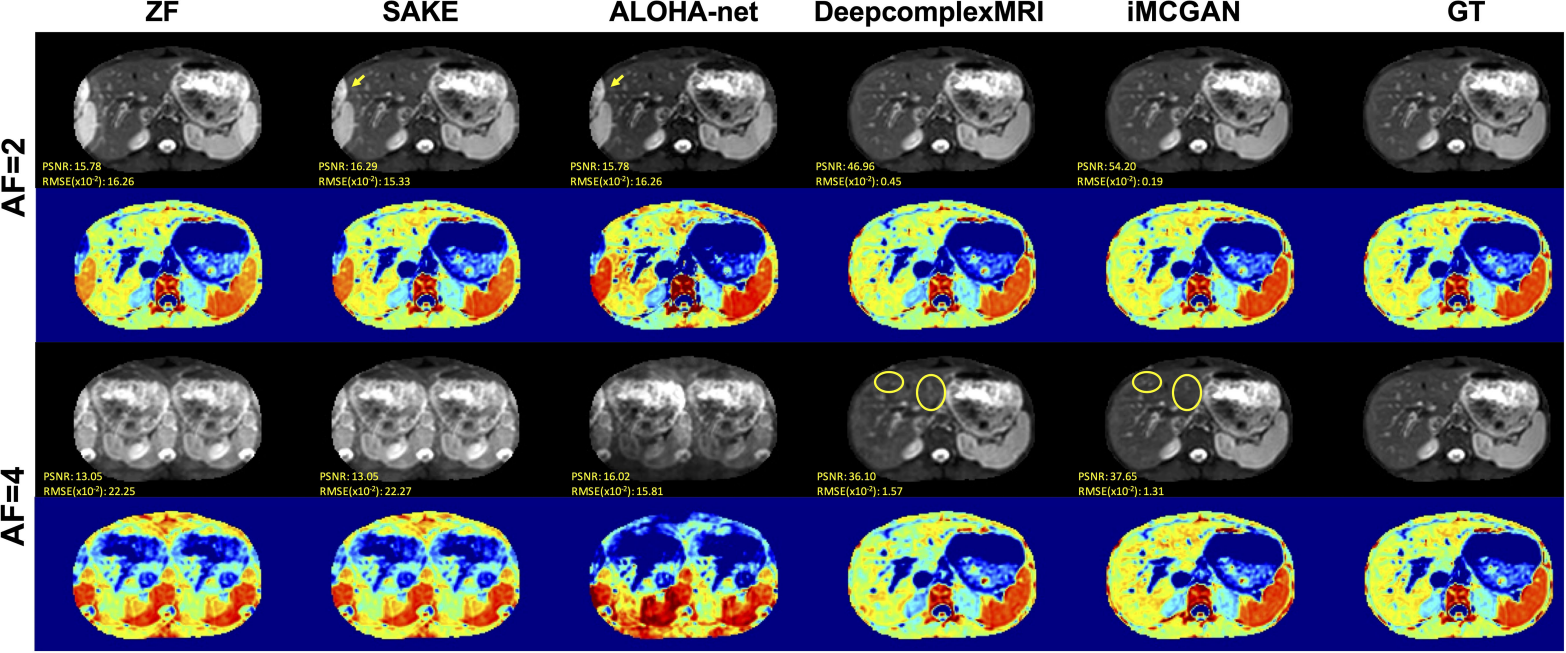
The visual comparison between several advanced DWI (b = 800 s/mm^2^) reconstruction methods under different AFs. The first and third row shows the reconstructed DWI with corresponding PSNR and SSIM values, and the second and fourth row shows the corresponding ADC maps.

Representative DWI reconstruction using different methods, and the ADC maps constructed from the reconstructed data and corresponding ground truth, are shown in **Figure 7**. The images reconstructed by SAKE and ALOHA-net exhibited aliasing artifacts on the liver when the AF was 2 as indicated by the yellow arrows. In contrast, the DeepcomplexMRI and iMCGAN reconstruction were free of artifacts. As the AF increased, the liver structure was blurred in the reconstruction images using SAKE and ALOHA-net. In contrast, the model iMCGAN significantly improved the image quality by recovering vessels (as indicated by yellow circles) that were not visible by the other reconstruction methods. Furthermore, the iMCGAN outperformed other methods significantly (*p<* 0.001). The ADC maps of the iMCGAN were also superior to those obtained using the other methods.

**Figure 8:**
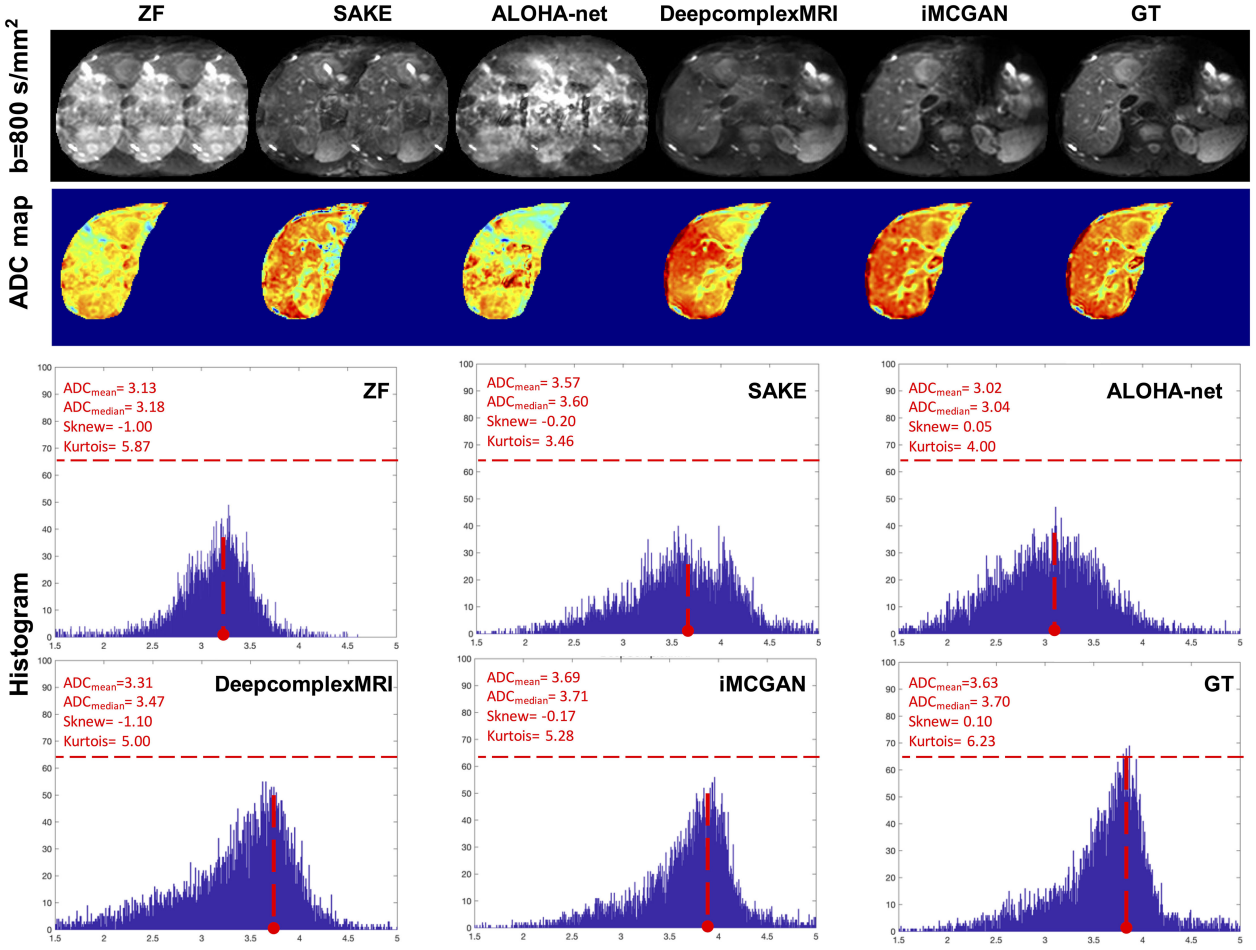
The representative reconstructed DWI (b=800 s/mm^2^) of the tumor at the AF of 4. The second to fourth rows show the whole-liver ADC maps and the corresponding histograms of the tumor ADC maps. The histogram parameters are displayed in the upper left corner.

Representative reconstructed DWI (b = 800 s/mm^2^) of tumors using different methods at the AF of 4 are shown in **Figure 8**. The image reconstructed using SAKE and ALOHA-net exhibited ghosting artifacts, which obscured the tumor characteristics. The iMCGAN and DeepcomplexMRI greatly improved the ZF image quality by recovering sharpness and adding more structural details to the ZF images. The quantitative indicators from the histogram showed that iMCGAN (ADCmean = 3.69; ADCmedian = 3.71, Sknew = –0.17, kurtosis = 5.28) achieved the closest quantifications to the GT (ADCmean = 3.63; ADCmedian = 3.70; Sknew = 0.10; kurtosis = 6.23) as compared to other methods.

**Figure 9:**
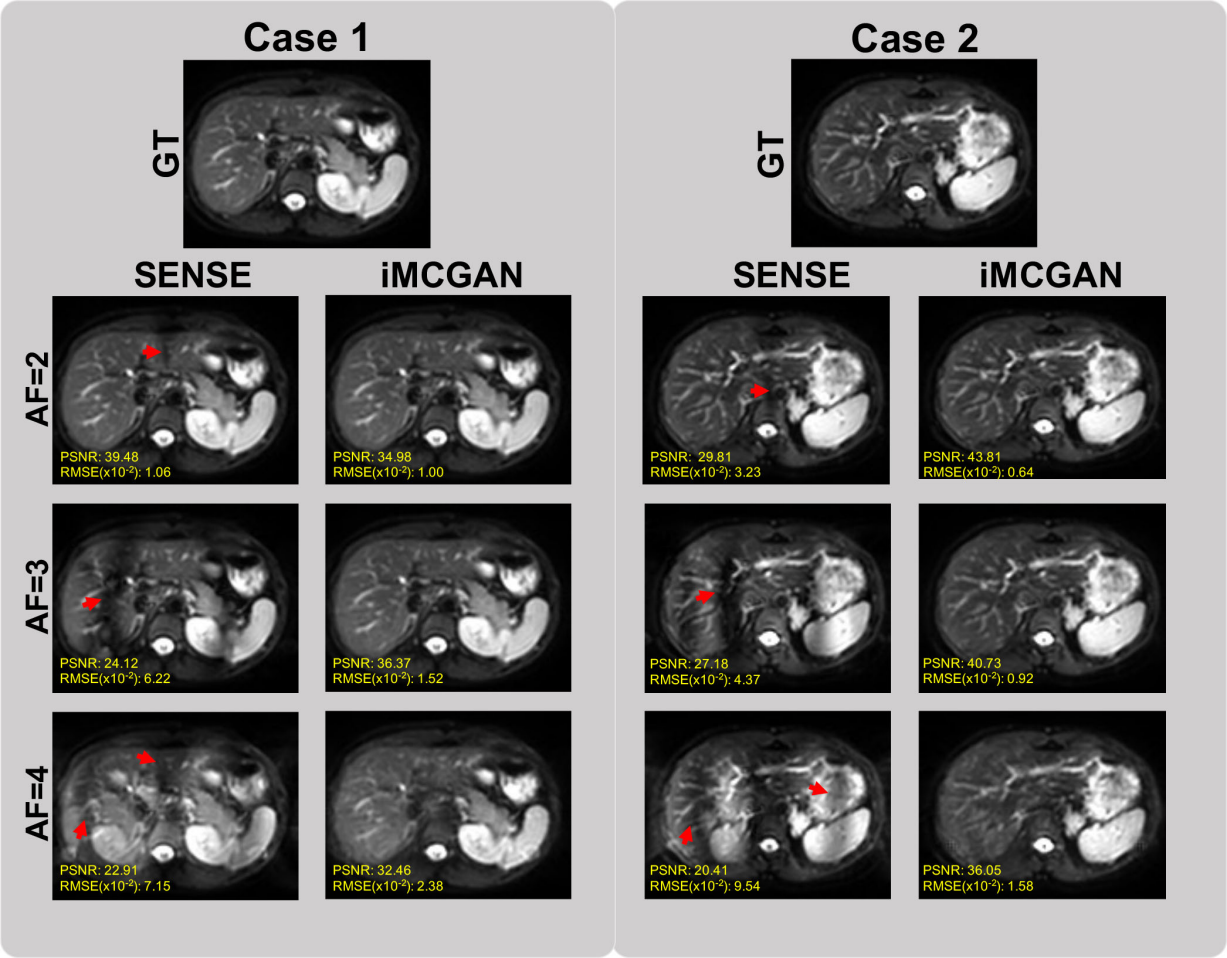
The comparisons between the SENSE and iMCGAN reconstructions with the Afs of 2, 3, and 4. All images reconstructed with the proposed iMCGAN were free of aliasing artifacts.

**Figure 10:**
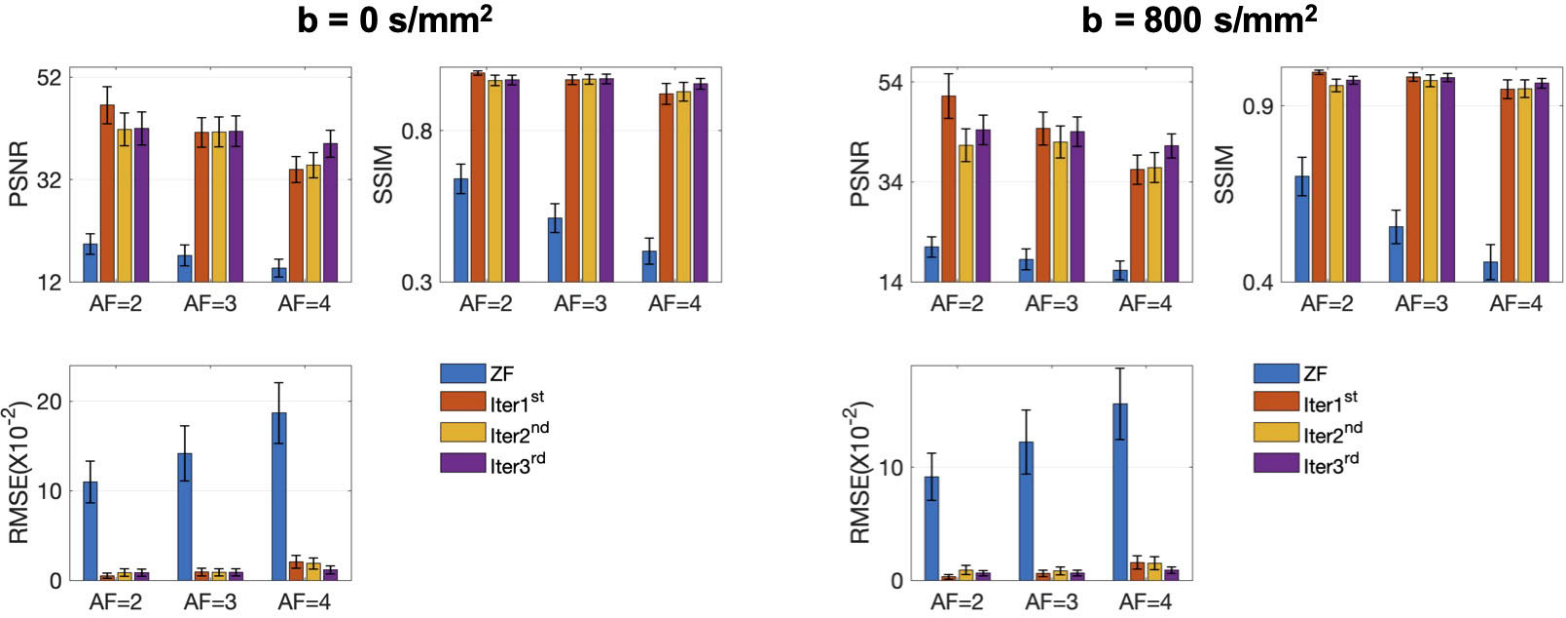
The quantitative metrics were calculated on the ZF, Iter 1st, Iter 2nd, and Iter 3rd reconstructions at different AFs. The error bars on each series indicate the standard deviation of each quantitative variable.

The conventional SENSE and the proposed iMCGAN for two participants at different AFs were compared (**Figure 9**). When the AF was 2, some ghosting artifacts (red arrows) were observed in the SENSE reconstructions due to the mismatch between the DWI image and the sensitivity map. However, such kinds of artifacts were eliminated using the proposed iMCGAN. As the AF increased, the SENSE reconstruction gradually worsened and the performance differences between the iMCGAN and SENSE became larger. These ghosting artifacts severely affected the estimation of ADC maps. Residual aliasing artifacts were not noticeable on the images reconstructed using the proposed method. Iter 1st (PSNR: 46.55 ± 3.63; SSIM: 0.99 ± 0.01; NMSE: 0.52 ± 0.32) showed the best results when the AF was 2 for b = 0 images (**Figure 10A**). Iter 3rd (PSNR: 41.46 ± 3.00; NMSE: 0.91 ± 0.42) significantly increased the PSNR and decreased the RMSE compared with other two iterations when the AFs were 3 and 4. The metrics of Iter 1st showed the optimal results when the AFs were 2 and 3 for b = 800 images (**Figure 10B**). Iter 3rd (PSNR: 41.24 ± 2.4, SSIM: 0.96 ± 0.03, NMSE: 0.90 ± 0.28) obviously increased the PSNR and SSIM and decreased the RMSE compared with other two iterations when the AF was 4. The quantitative metrics of different models on b = 0 s/mm^2^ and 800 s/mm^2^ images at the AF of 2, 3, and 4 are shown in **Table 2**. The proposed iMCGAN resulted in the best performance in terms of the greatest PSNR (41.82 ± 2.14), least RMSE (0.90 ± 0.28), and highest SSIM (0.96 ± 0.03) for b = 800 s/mm^2^ DWI at the AF of 4.

**Table 2:** The PSNR, SSIM and RMSE of different reconstruction methods with the AF of 2, 3, and 4.

b = 0 s/mm^2^ DWI									
	*AF = 2*			*AF = 3*			*AF = 4*		
	PSNR	SSIM	RMSE	PSNR	SSIM	RMSE	PSNR	SSIM	RMSE
ZF	20.47 ± 1.82	0.76 ± 0.03	9.67 ± 1.86	19.34 ± 2.05	0.69 ± 0.06	11.08 ± 2.46	16.41 ± 1.57	0.64 ± 0.05	15.37 ± 2.71
SAKE	20.69 ± 1.95	0.75 ± 0.06	9.46 ± 1.99	19.77 ± 2.07	0.66 ± 0.06	10.55 ± 2.34	17.4 ± 1.96	0.65 ± 0.05	13.82 ± 3
ALOHA-net	22.07 ± 2.01	0.44 ± 0.07	8.08 ± 1.73	20.2 ± 1.72	0.33 ± 0.06	9.96 ± 1.84	19.59 ± 1.57	0.3 ± 0.04	10.65 ± 1.9
DeepcomplexMRI	41.98 ± 3.52	0.97 ± 0.02	0.90 ± 0.41	39.30 ± 3.17	0.97 ± 0.02	0.94 ± 0.42	38.68 ± 2.37	0.95 ± 0.02	1.53 ± 1.08
iMCGAN	**42.00 ± 3.21**	**0.97 ± 0.02**	**0.86 ± 0.41**	**41.46 ± 3.00**	**0.97 ± 0.02**	**0.91 ± 0.42**	**39.80 ± 2.69**	**0.95 ± 0.02**	**1.18 ± 0.45**
b = 800 s/mm^2^									
	*AF=2*			*AF=3*			*AF=4*		
	PSNR	SSIM	RMSE	PSNR	SSIM	RMSE	PSNR	SSIM	RMSE
ZF	21.41 ± 1.98	0.77 ± 0.03	8.71 ± 1.87	20.53 ± 1.90	0.74 ± 0.04	9.63 ± 2.04	17.01 ± 1.69	0.69 ± 0.04	14.37 ± 2.72
SAKE	21.61 ± 2.09	0.79 ± 0.05	8.54 ± 1.97	20.63 ± 1.78	0.71 ± 0.06	9.52 ± 2.05	17.38 ± 1.78	0.69 ± 0.05	13.81 ± 2.73
ALOHA-net	23.70 ± 2.10	0.45 ± 0.07	6.72 ± 1.56	20.76 ± 2.11	0.30 ± 0.08	9.39 ± 2.03	20.43 ± 2.11	0.21 ± 0.06	9.79 ± 2.26
DeepcomplexMRI	49.45 ± 4.55	0.98 ± 0.01	0.49 ± 0.30	43.84 ± 3.31	0.98 ± 0.01	0.74 ± 0.30	39.78 ± 2.78	0.95 ± 0.02	1.09 ± 0.43
iMCGAN	**51.22 ± 4.47**	**0.99 ± 0.01**	**0.32 ± 0.22**	**44.7 ± 3.26**	**0.98 ± 0.01**	**0.63 ± 0.29**	**41.82 ± 2.14**	**0.96 ± 0.03**	**0.90 ± 0.28**

The numbers in bold indicates the best performance.

The quantitative metrics of different models on b = 0 and 800 images at the AF of 2, 3, and 4 are shown in **Table 2**. The proposed iMCGAN resulted in the best performance in terms of the greatest PSNR (41.82 ± 2.14), least RMSE (0.90 ± 0.28), and highest SSIM (0.96 ± 0.03) for b = 800 DWI at the AF of 4.

## 5 Discussion

This study constructed an iterative multi-channel GAN-based framework for simultaneous sensitivity estimation and reconstruction. The iMCGAN model showed two advantages: (1) The architecture iteratively refined the sensitivity maps and the multi-channel reconstructed images. Thus, the quality of reconstruction was improved, and the aliasing artifact was reduced. (2) The sensitivity maps obtained from the data effectively eliminated the mismatch between coil calibration scan and main scan due to the interscan motion.

The image quality and quantitative parameters for both b = 0 s/mm^2^ and 800 s/mm^2^ reconstructions were improved with the increasing number of iterations at a high AF (AF = 4). However, the parameters from b = 0 s/mm^2^ and 800 s/mm^2^ reconstructions were not improved as the number of iterations increased at a lower AF (AF = 2). This outcome might have been because one iteration was already satisfied when the AF was small and the model converged more quickly. Also, at the AF of 3, the best outcome was obtained for b = 0 s/mm^2^ at the 3rd iteration and b = 800 s/mm^2^ at the 1st iteration. The edge characteristics on the image presented high intensities when b = 0 s/mm^2^ and it was difficult to recover the edge signal when the images were aliased together. When the AF is low, only one iteration is required. More iterations are recommended when the AF becomes higher.

It is observed that iMCGAN model trained only with healthy images performed well on images with tumors. These can be explained by that the iMCGAN contains the data-fidelity term, which guarantees the consistency of the reconstruction with the acquired data.

The iMCGAN model outperformed SAKE, ALOHA-net, and DeepcomplexMRI methods at different AFs. The iMCGAN significantly increased the PSNR and SSIM and lowered the RMSE compared with other reconstruction methods. The PI based on EPI sequences usually requires uniform sampling. Therefore, it is difficult to estimate the true underlying coil sensitivity maps. The SAKE reconstruction depends on the low-rank recovery and requires manual tuning parameters. Further, the computational complexity of the SAKE is large. The ALOHA-net learns *k*-space interpolation kernels in an end-to-end fashion from the *k*-space to the image domain using mean square error loss. However, it is not fitting for the under-sampled multi-channel DWI reconstruction without the ACS line. The DeepcomplexMRI is also end-to-end learning without explicitly using coil sensitivity maps to recover the channel-wise images. When the AF increases, the iterative approach is better than the direct reconstruction approach. Nevertheless, the iMCGAN model, a learning-based method, benefits from large amounts of previously acquired data. Hence, this framework performs better in acquiring and preserving the structure and details of the reconstructed images. Many previous studies Shaul et al. (39); Murugesan et al. (40); Liu et al. (41) proved that GAN reconstructed significantly better compared with plain CNN architecture in terms of the PSNR, SSIM, and RMSE.

The single-shot EPI had the drawbacks of low spatial resolution and signal-to-noise ratio Ni et al. (42). The multi-shot EPI technique addresses this drawback. Segmented EPI Wu and Miller (43) separates the *k*-space in an interwoven manner along the phase encoding direction. Since the traverse of the *k*-space is accelerated at each shot, segmented EPI has higher resolution and SNR and lower distortion compared with the single-shot EPI. However, shot-to-shot phase changes due to the patient’s motion severely degrade the image quality. Therefore, segmented EPI is not yet widely used for abdominal DW imaging Lewis et al. (44). The iMCGAN model could avoid the inconsistency of the sensitivity maps and DW data caused by respiratory motion.

This current model used a different architecture compared with the previous method, MLP Kwon et al. (28). All voxels in the multi-channel aliased images along the phase encoding direction were used as the input of the network in the MLP. The MLP is also a fully connected network and is more computationally expensive to be trained than the iMCGAN model. Besides, the MLP uses vector as input while the iMCGAN uses matrix as input. Therefore, the iMCGAN can extract the spatial relation better between pixels of images. In addition, the other two previous methods Zhang et al. (29); Wang et al. (34) estimated missing data directly with the trained network. However, the iMCGAN not only calculated the sensitivity maps but also optimized the DW image iteratively., The first iteration of the process is the most effective in removing artifacts and the next two iterations refine them. The number of GANs must be an integer multiple of 2 for each iteration, one of them is used to learn the coil sensitivity map and the other is used to learn the reconstruction of the image. Therefore, each network performs a different task, and a single deep GAN without iteration canâ€™t carry out an acceptable reconstruction.

This study had several limitations. First, the improvement of the generalization performance of the iMCGAN is required by tuning our network according to the number of coils from each scanner. Like other deep learning-based methods, the iMCGAN should be retrained for the dataset using different channel numbers. Several networks can be trained with a certain number of channels. The appropriate network can be selected to reconstruct the unseen under-sampled data using different multi-channel. Second, the image reconstruction performance can be improved by introducing some edge- or texture-preserved regulations into the loss function of the network. Third, further studies are needed to assess the performance of the proposed iMCGAN in patients with other diseases. Besides, data collection from different devices, field strengths, and different anatomies should be considered.

## 6 Conclusions

The study constructed a novel calibration-free sensitivity estimation and image reconstruction framework for under-sampled multi-channel liver DWI. The performance was validated on healthy volunteers and patients with tumors. The quality of the reconstructed image was improved, and the aliasing artifact was alleviated when motions occurred during the imaging procedure.

## Data availability statement

The original contributions presented in the study are included in the article/supplementary material. Further inquiries can be directed to the corresponding authors.

## Ethics statement

The studies involving human participants were reviewed and approved by Ruijin Hospital. The patients/participants provided their written informed consent to participate in this study.

## Author contributions

Conceptualization, JL and CW; methodology, JL; validation, YL and FY; investigation, JL and CW; writing—original draft preparation, JL; writing—review and editing, WC; visualization, RL; supervision, RL; funding acquisition, JL and CW. All authors contributed to the article and approved the submitted version.
